# Effects of the proportion of high-risk patients and control strategies on the prevalence of methicillin-resistant *Staphylococcus aureus* in an intensive care unit

**DOI:** 10.1186/s12879-019-4632-9

**Published:** 2019-12-03

**Authors:** Farida Chamchod, Prasit Palittapongarnpim

**Affiliations:** 1 Department of Mathematics, Faculty of Science, Mahidol University, Bangkok, Thailand; 2 Department of Microbiology, Faculty of Science, Mahidol University, Bangkok, Thailand

**Keywords:** MRSA transmission, Infection prevention and control, Targeted control strategies, Sensitivity analysis

## Abstract

**Background:**

The presence of nosocomial pathogens in many intensive care units poses a threat to patients and public health worldwide. Methicillin-resistant *Staphylococcus aureus* (MRSA) is an important pathogen endemic in many hospital settings. Patients who are colonized with MRSA may develop an infection that can complicate their prior illness.

**Methods:**

A mathematical model to describe transmission dynamics of MRSA among high-risk and low-risk patients in an intensive care unit (ICU) via hands of health care workers is developed. We aim to explore the effects of the proportion of high-risk patients, the admission proportions of colonized and infected patients, the probability of developing an MRSA infection, and control strategies on MRSA prevalence among patients.

**Results:**

The increasing proportion of colonized and infected patients at admission, along with the higher proportion of high-risk patients in an ICU, may significantly increase MRSA prevalence. In addition, the prevalence becomes higher if patients in the high-risk group are more likely to develop an MRSA infection. Our results also suggest that additional infection prevention and control measures targeting high-risk patients may considerably help reduce MRSA prevalence as compared to those targeting low-risk patients.

**Conclusions:**

The proportion of high-risk patients and the proportion of colonized and infected patients in the high-risk group at admission may play an important role on MRSA prevalence. Control strategies targeting high-risk patients may help reduce MRSA prevalence.

## Background

Nosocomial infections continue to be a major burden globally causing morbidity and mortality in patients, and increasing additional costs to patients and healthcare providers [1, 2]. Those infections are caused by many types of microorganisms including gram-positive bacteria like *Staphylococcus aureus* (*S. aureus*) [1, 3]. Generally, *Staphylococcus aureus* can cause wound infections in patients but it can be life-threatening if it spreads to the lungs, the bloodstream, and other organs. Approximately, 10% to 40% of normal people carry *S. aureus*, including both methicillin-sensitive and resistant *S. aureus* (MSSA and MRSA), in their interior nares [4]. Although only 1% to 2% of them carry MRSA, it accounts for more than 60% of hospital-onset *S. aureus* infections [5, 6]. MRSA is known to have developed resistance to several widely used antibiotics; resistance to newer antimicrobial agents such as linezolid, vancomycin, teicoplanin, and daptomycin has also been reported [7]. Although rates of infection with MRSA have slowly declined in recent years, the disease risk still remains substantial and it is even more threatening when only limited numbers of antimicrobial agents are currently in develoment [8, 9].

In a hospital setting, one of the most common places of MRSA colonization and infection is an intensive care unit (ICU) [10–12]. It serves as a reservoir for dissemination of colonized and infected patients in the hospital [13]. Approximately, 20% of infected patients will die from invasive MRSA infections in ICUs; therefore, it is very important to be able to identify the risk factors for colonization and active infection [14]. Risk factors for MRSA colonization and infection have been investigated in numerous studies[14–18]. MRSA colonization itself is the most important risk factor for active infection [19]. Patients with certain comorbidities such as diabetes mellitus and chronic pulmonary disease have been reported to be at risk of MRSA infections [17, 20]. Preceding studies suggest that advanced age and patient demographics such as residence of a nursing home are also associated with MRSA colonization and infection [21, 22]. Other factors include prolonged hospitalization, exposure to invasive equipment or procedures, the presence of colonized or infected patients in the same area at the same time, previous hospitalization, and exposure to antibiotics [20, 23].

Infection prevention and control is a fundamental key to prevent and reduce MRSA transmission in health care settings. Usually, control measures that have been implemented in hospitals vary widely and have demonstrated various degrees of success [24, 25]. Basic control recommendations often include hand hygiene practice, proper cleaning and disinfection of equipment and environment, implementation of a monitoring program, and contact precautions for MRSA colonized and infected patients [26, 27]. Note that the latter recommendation requires that colonized patients being placed in single or private rooms [27]. If rooms are not available, cohorting of patients is acceptable. In addition, if there are opportunities for improvement, hospitals may consider adopting other prevention approaches such as active surveillance for MRSA colonization and infection, implementing MRSA decolonization therapy, or implementing universal gowns and gloves [26, 27]. Although several preceding studies promote universal approaches, the merits of them are debated as some recommendations such as contact precautions or screening can be resource-intensive and costly [27, 28]. Consequently, alternative approaches such as enhanced hand-hygiene compliance and more targeted control strategies such as the use of decolonization therapy with high-risk patients and targeted screening are sometimes implemented [28, 29].

Mathematical modeling is one of the important tools to investigate the spread of MRSA among patients in many studies [30, 31, 33–36]. It can be used to understand the role influenced by different factors and the impact of implemented interventions. To our knowledge, in the deterministic framework, none of previous models have differentiated patients according to their risk of developing an MRSA infection, the probability of having unsuccessful treatment, and the probability of patients staying longer in an ICU. In this study, a mathematical model that patients are categorized into high-risk or low-risk groups was developed to investigate the impact of high-risk patients and control strategies on MRSA prevalence.

## Methods

### Model formulation

Within an ICU, patients are divided into two groups: high-risk and low-risk. In this work, high-risk patients are those who are more likely to be colonized with MRSA, have higher risks of developing an MRSA infection, stay in a facility longer, and are more likely to die from the infection. Low-risk patients are those who are not high-risk patients. Each group consists of three mutually exclusive classes: uncolonized (*U*_*i*_), colonized (*C*_*i*_), and infected (*I*_*i*_), for *i*=*H*,*L*. Note that the subscript *i* describes a group that patients belong to, either the high-risk group (*H*) or the low-risk group (*L*). Hence, the total number of patients in the ICU (*N*_*p*_) is $\sum _{i=H,L}U_{i}+C_{i}+I_{i}$. For simplicity, it is assumed that there are no transitions between two groups of patients. HCWs are categorized into two classes: uncontaminated (*H*) and transiently contaminated (*H*_*C*_). The total number of HCWs (*N*_*h*_) is given by *H*+*H*_*C*_. To describe state movements of patients and HCWs in the compartmental model, the following assumptions are made.

**Admissions.** Patients are admitted to the ICU at a total rate of *Λ* with the proportion *θ* of being in the high-risk group. In the high-risk group, the proportions *λ*_*CH*_ and *λ*_*IH*_ of patients are colonized and infected at admission, respectively. Moreover, for the low-risk group, the proportions *λ*_*CL*_ and *λ*_*IL*_ of patients are colonized and infected at admission. It is assumed that the total number of patients in the ICU remains constant so that the number of new admissions is equal to the number of patient discharges and deaths.

**Discharges.** Uncolonized patients in each group are discharged at a rate of *γ*_*i*_ for *i*=*H*,*L* where 1/*γ*_*i*_ is the average length of stay in the ICU of group *i*. Colonized patients are discharged at a rate of (1−*p*_*i*_)*γ*_*i*_ for *i*=*H*,*L* where *p*_*i*_ is the probability that colonized patients in group *i* develop an MRSA infection. Additionally, colonized patients in the high-risk group are assumed to be more likely to develop an infection (*p*_*H*_≥*p*_*L*_) and stay longer in the ICU (1/*γ*_*H*_<1/*γ*_*L*_). Infected patients are treated at a rate of *ν*_*i*_ for *i*=*H*,*L* where 1/*ν*_*i*_ is the average length of antimicrobial therapy. It is further assumed that it may take longer to treat infected patients in the high-risk group (*ν*_*L*_≥*ν*_*H*_). After treatment, infected patients either die from an MRSA infection with the probability *d*_*i*_ for *i*=*H*,*L* or become colonized patients again with the probability 1−*d*_*i*_. Due to the shorter length of stay (LOS) of patients in the ICU as compared to the length of natural clearance of bacteria, we assume that there are no movements of patients from the colonized and infected compartments to the uncolonized compartment. This assumption is similar to one made in other previous studies [37, 38].

**MRSA transmission.** Hands of HCWs are important vehicles for MRSA transmission from one patient to another. Here, transmission of MRSA from contaminated hands of HCWs to uncolonized patients in each group occurs at a rate of $\frac {\beta }{N_{p}}H_{c}U_{i}$, for *i*=*H*,*L*, where *β* is a transmission coefficient from a contaminated HCW to uncolonized patients. Such a term is based on an assumption that the probability of successful colonization and the average contact number between HCWs and patients are not different between high-risk and low-risk patients. Hands of uncontaminated HCWs can become contaminated with MRSA after contacting colonized or infected patients at a rate of $\frac {\alpha _{C}}{N_{p}}(C_{H}+C_{L})H + \frac {\alpha _{I}}{N_{p}}(I_{H}+I_{L})H$ where *α*_*C*_ and *α*_*I*_ represent transmission coefficients of MRSA from colonized and infected patients to an uncontaminated HCW respectively, with *α*_*I*_>*α*_*C*_. In those terms, the probability of successful contamination in a HCW is assumed to be different during HCWs contacting colonized and infected patients.

**Infection prevention and control.** Standard control measures such as effective hand-washing are taken into account via the average time that HCWs stay contaminated (1/*η*) and the probabilities of successful colonization and contamination (*q*,*q*_*C*_, and *q*_*I*_). If hand hygiene compliance is high (or equivalently 1/*η*,*q*,*q*_*C*_, and *q*_*I*_ are small), MRSA is unlikely to be transmitted among patients. Two parameters that particularly reflect additional and targeted measures to control the spread of MRSA among patients include a reduction term for infected patients (1−*κ*,0≤*κ*≤1) and another reduction term for high-risk patients (1−*σ*,0≤*σ*≤1). If those targeted measures towards high-risk patients completely help prevent MRSA transmission, we have *σ*=1. Similarly, if those targeted measures towards infected patients entirely prevent transmission of MRSA, then we have *κ*=1. All the transmission terms after incorporating additional and targeted control factors become $(1-\sigma)\frac {\beta }{N_{p}}H_{C}U_{H}, (1-\sigma)\frac {\alpha _{C}}{N_{p}}C_{H}H$, and $(1-\kappa)\frac {\alpha _{I}}{N_{p}}((1-\sigma)I_{H}+I_{L})H$.

**Fig. 1:**
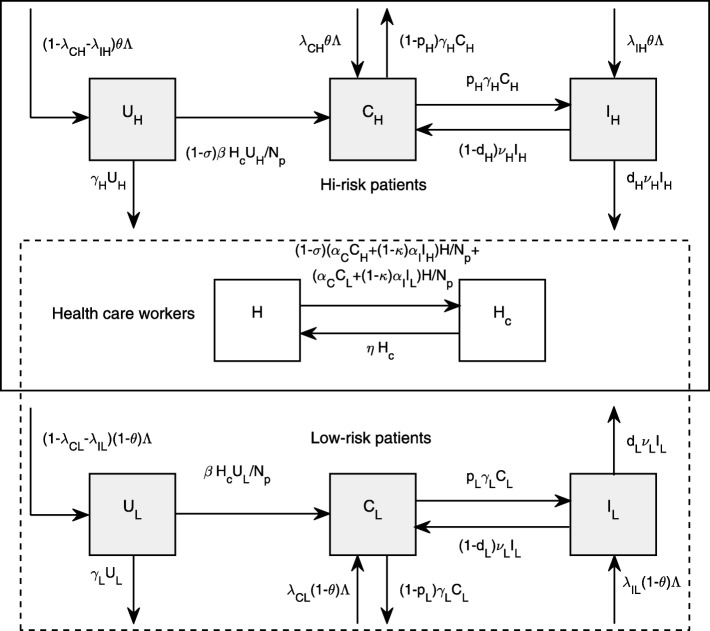
Flow diagram. A flow diagram for describing MRSA transmission dynamics among high-risk and low-risk patient groups via hands of HCWs. Each group of patients is divided into three categories: susceptible (*S*_*i*_), colonized (*C*_*i*_), and infected (*I*_*i*_) for *i*=*H*,*L*

From the aforementioned assumptions, a model for describing transmission dynamics of MRSA among high-risk and low-risk patients via hands of HCWs is described by

1 $$ {}{\begin{array}{lcl} \frac{dU_{H}}{dt} & = & (1-\lambda_{CH}-\lambda_{IH})\theta\Lambda- (1-\sigma)\frac{\beta}{N_{p}}H_{C}U_{H}-\gamma_{H}U_{H},\\ \frac{dC_{H}}{dt} & = & \lambda_{CH}\theta\Lambda+(1-d_{H})\nu_{H}I_{H}+ (1-\sigma)\frac{\beta}{N_{p}}H_{C}U_{H}-\gamma_{H}C_{H},\\ \frac{dI_{H}}{dt} & = & \lambda_{IH}\theta\Lambda+p_{H}\gamma_{H}C_{H}-\nu_{H}I_{H},\\ \frac{dU_{L}}{dt} & = & (1-\lambda_{CL}-\lambda_{IL})(1-\theta)\Lambda-\frac{\beta}{N_{p}}H_{C}U_{L}-\gamma_{L}U_{L},\\ \frac{dC_{L}}{dt} & = & \lambda_{CL}(1-\theta)\Lambda+(1-d_{L})\nu_{L}I_{L}+\frac{\beta}{N_{p}}H_{C}U_{L}-\gamma_{L}C_{L},\\ \frac{dI_{L}}{dt} & = & \lambda_{IL}(1-\theta)\Lambda+p_{L}\gamma_{L}C_{L}-\nu_{L}I_{L},\\ \frac{dH}{dt} & = & \eta H_{C}-\frac{\alpha_{C}}{N_{p}}((1-\sigma)C_{H}+C_{L})H -(1-\kappa)\frac{\alpha_{I}}{N_{p}}((1-\sigma)I_{H}+I_{L})H,\\ \frac{dH_{C}}{dt} & = & \frac{\alpha_{C}}{N_{p}}((1-\sigma)C_{H}+C_{L})H+ (1-\kappa)\frac{\alpha_{I}}{N_{p}}((1-\sigma)I_{H}+I_{L})H-\eta H_{C}, \end{array}}  $$

with *Λ*=(*γ*_*H*_*U*_*H*_+(1−*p*_*H*_)*γ*_*H*_*C*_*H*_+*d*_*H*_*ν*_*H*_*I*_*H*_)+(*γ*_*L*_*U*_*L*_+(1−*p*_*L*_)*γ*_*L*_*C*_*L*_+*d*_*L*_*ν*_*L*_*I*_*L*_). A flow diagram for describing the compartmental model is illustrated in Fig. 1 and parameter descriptions are summarized in Table 1.

**Table 1:** List of parameters for MRSA transmission among high-risk and low-risk patients via HCWs

Description	Symbol(s)	Value(s)	References
Proportion of high-risk patients	*θ*	0.5,[0,1]	[39]
Proportions of colonized patients in the high-risk and low-risk groups at admission	*λ*_*CH*_,*λ*_*CL*_	0.2, 0.05	[40]
Proportions of infected patients in the high-risk and low-risk groups at admission	*λ*_*IH*_,*λ*_*IL*_	0.01, 0	[41]
Discharge rates of uncolonized and colonized patients in the	*γ*_*H*_,*γ*_*L*_ high-risk and low-risk groups	1/9,1/5	[29, 42, 43]
Probabilities of developing an MRSA infection in high-risk and low-risk patients	*p*_*H*_,*p*_*L*_	0.2, 0.05	[41, 44]
Treatment rates for high-risk and low-risk patients	*ν*_*H*_,*ν*_*L*_	1/10,1/5	[45]
Probabilities of MRSA-related death in high-risk and low-risk patients	*d*_*H*_,*d*_*L*_	0.3, 0.15	[14, 39, 46]
Total numbers of patients	*N*_*p*_	48	
Ratio of patients to HCWs	*r*	4	[39]
Decontamination rate in HCWs	*η*	24	[13]
Per capita contact rate	*c*	1.38	[30]
Probability of successful colonization	*q*	0.01	[13, 31]
Probability of successful contamination for colonized and infected patients	*q*_*C*_,*q*_*I*_	0.17, 0.25	[13, 32]
Transmission coefficient from contaminated HCWs to uncolonized patients	*β*	*cqN*_*p*_	
Transmission coefficients from colonized and infected patients to uncontaminated HCWs	*α*_*C*_,*α*_*I*_	*cq*_*C*_*N*_*p*_,*cq*_*I*_*N*_*p*_	
Control factor for targeted measures on high-risk patients	*σ*	0.5,[0,1]	varying
Control factor for targeted measures on infected patients	*κ*	0.5,[0,1]	varying

### The basic reproduction number

For a special case, when there are no admissions of colonized and infected patients in both groups in the ICU, *λ*_*CH*_=*λ*_*IH*_=*λ*_*CL*_=*λ*_*IL*_=0, there is an MRSA-free steady state of (1) and it is given by

$$\begin{array}{lcl} E^{0} & = & (U_{H}^{0},C_{H}^{0},I_{H}^{0},U_{L}^{0},C_{L}^{0},I_{L}^{0},H^{0},H_{C}^{0})\\ & = & \left(\frac{\theta\gamma_{L}N_{p}}{[(1-\theta)\gamma_{H}+\theta\gamma_{L}]},0,0,\frac{(1-\theta)\gamma_{H}N_{p}}{[(1-\theta)\gamma_{H}+\theta\gamma_{L}]},0,0,N_{h},0\right). \end{array}$$

Then, the basic reproduction number can be calculated by the next-generation matrix. Two matrices, **F** and **V**, can be obtained from the Jacobian matrices at the MRSA-free steady state *E*^0^ of the $\mathcal {F}$-matrix describing new infections and the $\mathcal {V}$-matrix describing compartmental movements [48]:

$${}{\mathbf{F}=\left[\begin{array}{ccccc} 0 & 0 & \frac{(1-\sigma)\beta}{N_{p}}U_{H}^{0} & 0 & 0\\ 0 & 0 & \frac{\beta}{N_{p}}U_{L}^{0} & 0 & 0\\ \frac{(1-\sigma)\alpha_{C}}{N_{p}}H^{0} & \frac{\alpha_{C}}{N_{p}}H^{0} & 0 &\frac{(1-\kappa)(1-\sigma)\alpha_{I}}{N_{p}}H^{0} & \frac{(1-\kappa)\alpha_{I}}{N_{p}}H^{0}\\ 0 & 0 & 0 & 0 & 0\\ 0 & 0 & 0 & 0 & 0 \end{array}\right] \text{ and}}$$

$${}{\mathbf{V}=\left[\begin{array}{ccccc} \gamma_{H} & 0 & 0 & -(1-d_{H})\nu_{H} & 0\\ 0 & \gamma_{L} & 0 & 0 & -(1-d_{L})\nu_{L}\\ 0 & 0 & \eta & 0 & 0\\ -p_{H}\gamma_{H} & 0 & 0 & \nu_{H} & 0\\ 0 & -p_{L}\gamma_{L} & 0 & 0 & \nu_{L} \end{array}\right].}$$

The basic reproduction number is defined as the spectral radius of **FV**^−1^:

2 $$ \begin{array}{lcl} R_{0}^{2} & = &\frac{(1-\sigma)^{2}\beta\alpha_{C}}{\eta(1-(1-d_{H})p_{H})\gamma_{H}} \frac{\theta\gamma_{L}}{[(1-\theta)\gamma_{H}+\theta\gamma_{L}]}\frac{N_{h}}{N_{p}}\\&+& \frac{(1-\sigma)^{2}(1-\kappa)p_{H}\beta\alpha_{I}}{\eta(1-(1-d_{H})p_{H})\nu_{H}}\frac{\theta\gamma_{L}}{[(1-\theta)\gamma_{H}+\theta\gamma_{L}]}\frac{N_{h}}{N_{p}}+\\ & & \frac{\beta\alpha_{C}}{\eta(1-(1-d_{L})p_{L})\gamma_{L}} \frac{(1-\theta)\gamma_{H}}{[(1-\theta)\gamma_{H}+\theta\gamma_{L}]}\frac{N_{h}}{N_{p}}\\&+& \frac{(1-\kappa) p_{L}\beta\alpha_{I}}{\eta(1-(1-d_{L})p_{L})\nu_{L}}\frac{(1-\theta)\gamma_{H}}{[(1-\theta)\gamma_{H}+\theta\gamma_{L}]}\frac{N_{h}}{N_{p}}. \end{array}  $$

Hence, when there are no admissions of colonized and infected patients in the ICU, MRSA dies out from the patient population if *R*_0_<1 and it persists among patients if *R*_0_>1.

As admissions of colonized patients and discharges of patients are generally present in the ICU, the above case may not be possible in reality. MRSA may persist even if *R*_0_<1 when there are admissions of colonized or infected patients. Note that when *λ*_*CH*_≠0 and *λ*_*CL*_≠0, there exists only a disease-present steady state of (1). This can be proved by contradiction and it is omitted here. Due to several nonlinear terms in the model, calculating the disease-present steady state (*E*^∗^) explicitly is not possible. Hence, the effects of certain parameters in the model on the prevalence of MRSA colonization or infection are numerically investigated.

### Sensitivity analysis

To identify important parameters that significantly influence MRSA prevalence in the ICU, sensitivity indices at the endemic steady state to the model parameters are calculated. We follow the steps in [49] to calculate the indices. The normalized forward sensitivity index of a variable *X*_*i*_ with respect to a parameter *p*_*i*_ is defined by

$$\omega_{p_{i}}^{X_{i}}=\frac{\partial X_{i}}{\partial p_{i}}\frac{p_{i}}{X_{i}}.$$

Since there are eight state variables at the endemic steady state (*E*^∗^) and twenty parameters in the model, for ease of notation (*U*_*H*_,*C*_*H*_,…,*H*_*C*_) is relabeled by (*X*_1_,*X*_2_,…,*X*_8_) and (*θ*,*λ*_*CH*_,…,*N*_*p*_) by (*p*_1_,*p*_2_,…,*p*_20_). In addition, the steady state Eq. (1) can be written as

$$f_{i}(X_{1},X_{2},\ldots,X_{8};p_{1},p_{2},\ldots,p_{20}) = 0 \;\text{ for} i=1,2,\ldots,8.$$

Differentiating both sides of the equations gives

$$\sum_{i=1}^{8}\frac{\partial f_{k}}{\partial X_{i}}\frac{\partial X_{i}}{\partial p_{j}}= -\frac{\partial f_{k}}{\partial p_{j}}.$$

for 1≤*k*≤8 and 1≤*j*≤20. Equivalently, the above system can be written in a matrix form as

$$JZ^{(j)}=B^{(j)}$$

where *J* is the Jacobian matrix of (1) at the endemic steady state and we have $Z^{(j)}=\left [\frac {\partial X_{i}}{\partial p_{j}}\right ]_{8\times 1}$, and $B^{(j)}=\left [-\frac {\partial f_{i}}{\partial p_{j}}\right ]_{8\times 1}$ for *i*=1,2,…8. Consequently, *Z*^(*j*)^ can be calculated and the sensitivity index of a variable *X*_*i*_ to the parameter *p*_*j*_ can be obtained by multiplying $\frac {\partial X_{i}}{\partial p_{j}}$ with $\frac {p_{j}}{X_{i}}$. By repeating the procedure with other parameters, sensitivity indices of state variables at the endemic steady state to the model parameters are obtained and shown in Table 2. The plus (minus) sign of a calculated value indicates that a state variable increases (decreases) when a parameter increases while the value reflects a magnitude of impact.

**Table 2:** Sensitivity indices of the state variables at the endemic steady state to the related parameters

	*U*_*H*_	*C*_*H*_	*I*_*H*_	*U*_*L*_	*C*_*L*_	*I*_*L*_	*H*	*H*_*C*_
High-risk group								
*θ*	+1.4147	-0.0006	-0.0006	-1.9996	-2.0023	-2.0010	+0.0018	-0.3578
*λ*_*CH*_	-0.6376	+1.2227	+1.0608	+0.2531	+0.2613	+0.2591	-0.0051	+1.0293
*λ*_*IH*_	-0.0406	+0.0466	+0.2140	+0.0127	+0.0132	+0.0130	-0.0003	+0.0632
*γ*_*H*_	-0.7898	+0.0019	+1.0064	+0.9999	+1.0024	+1.0015	-0.0016	+0.3160
*ν*_*H*_	+0.0814	-0.0002	-1.0002	0.0000	-0.0011	-0.0008	+0.0007	-0.1353
*d*_*H*_	+0.0313	-0.0840	-0.0699	0.0000	-0.0005	-0.0004	+0.0003	-0.0672
*p*_*H*_	-0.1284	+0.1632	+0.9675	0.0000	+0.0019	+0.0014	-0.0012	+0.2429
Low-risk group								
*λ*_*CL*_	-0.0036	+0.0003	+0.0003	-0.0527	+0.9766	+0.7107	-0.0009	+0.1722
*λ*_*IL*_	-0.0007	0.0000	0.0000	-0.0011	+0.0166	+0.2840	0.0000	+0.0053
*γ*_*L*_	+0.7050	-0.0003	-0.0003	-0.9996	-1.0089	-0.0065	+0.0009	-0.1717
*ν*_*L*_	+0.0026	0.0000	0.0000	0.0000	-0.0001	-1.0001	0.0000	-0.0086
*d*_*L*_	+0.0004	0.0000	0.0000	0.0000	-0.0105	-0.0040	0.0000	-0.0018
*p*_*L*_	-0.0036	0.0000	0.0000	0.0000	+0.0445	+0.7601	-0.0001	+0.0141
Others								
*σ*	+0.0017	-0.0032	-0.0028	-0.0003	-0.0069	-0.0052	+0.0041	-0.8254
*κ*	+0.0001	-0.0003	-0.0002	0.0000	-0.0011	-0.0008	+0.0007	-0.1439
*β*	-0.0010	+0.0018	+0.0015	0.0000	+0.0080	+0.0059	0.0000	+0.0028
*α*_*C*_	-0.0008	+0.0015	+0.0013	0.0000	+0.0068	+0.0051	-0.0043	+0.8590
*α*_*I*_	-0.0001	+0.0003	+0.0002	0.0000	+0.0011	+0.0008	-0.0007	+0.1439
*η*	+0.0010	-0.0018	-0.0015	0.0000	-0.0080	-0.0059	+0.0050	-1.0028
*N*_*p*_	+1.0018	-0.0033	-0.0029	+0.0001	-0.0149	-0.0110	+0.0043	-0.8738

The plus (minus) sign indicates that a state variable increases (decreases) when a parameter increases while the value reflects a magnitude of impact

## Results

To investigate the effects of certain parameters, including the admission proportions of colonized and infected patients in the high-risk and low-risk groups, the probability of developing an MRSA infection, and control factors on the prevalence of MRSA, numerical simulations are carried out. Based on the parameter values used in this study, the basic reproduction number is approximately 0.55. The approximation is obtained from the formula of *R*_0_ in (2) with the intermediate level of infection prevention and control towards high-risk and infected groups of patients (*σ*=*κ*=0.5). This leads to the overall prevalence of MRSA of 25% among patients and an acquisition rate of 10% in our baseline results. Note that here we define the acquisition rate as a percentage of patients who become colonized or infected with MRSA during their hospital stays. Hence, the quantity is approximately obtained from a subtraction of MRSA prevalence in the ICU and MRSA admission prevalence.

**Fig. 2:**
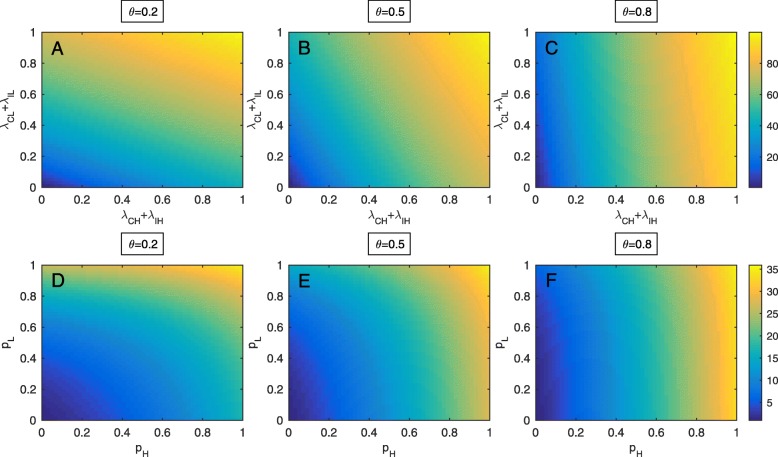
Effects of parameters on the prevalence of MRSA. **a**-**c** The prevalence of MRSA colonization and infection when the probabilities of colonization and infection at admission of high-risk (*λ*_*CH*_+*λ*_*IH*_) and low-risk patients (*λ*_*CL*_+*λ*_*IL*_) vary at three different levels of high-risk patients, low (*θ*=0.2), intermediate (*θ*=0.5) and high (*θ*=0.8). **d**-**f** The prevalence of MRSA infection according to the probabilities of becoming infected with MRSA of high- and low-risk patients at low, intermediate, and high levels of high-risk patients at admission

Figure 2a shows that when the proportion of high-risk patients at admission is small (*θ*=0.2), both of the admission proportions of colonized and infected patients in the high-risk and low-risk groups determine MRSA prevalence in the ICU. The higher proportions of colonized and infected patients in both groups at admission may result in the higher prevalence of MRSA. In Fig. 2b, when the proportion of high-risk patients at admission is intermediate (*θ*=0.5), the proportion of colonized and infected patients in the low-risk group at admission may only have a small impact on the prevalence of colonized and infected patients in the ICU. Changes in such term result in a smaller change of MRSA prevalence than changes in the proportion of colonized and infected patients in the high-risk group at admission. Such an impact becomes even smaller when the proportion of high-risk patients at admission becomes higher (*θ*=0.8)(see Fig. 2c). Figure 2d-f demonstrate the increasing prevalence of MRSA infection according to the increasing probabilities of developing infections of colonized patients in the high-risk and low-risk groups. When the proportion of high-risk patients is small in comparison to low-risk patients, both of the probabilities of developing infections in the high-risk and low-risk groups influence the prevalence of infected patients in the ICU. However, such an impact may decline resulting from the probability of becoming infected with MRSA in the low-risk group having a smaller effect when the proportion of high-risk patients in the ICU becomes higher (see Fig. 2e-f).

**Fig. 3:**
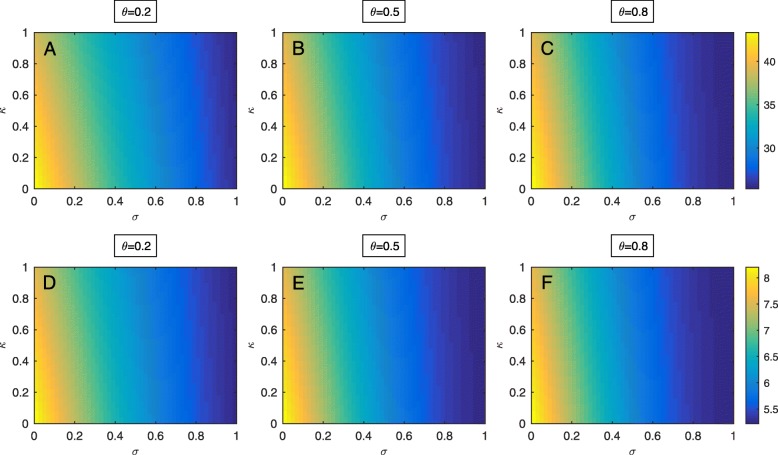
Control factors. Effects of changing control parameters related to control measures towards high-risk patients (*σ*) and infected patients (*κ*) on MRSA prevalence with three different proportions of high-risk patients in the ICU, low (*θ*=0.2), intermediate (*θ*=0.5) and high (*θ*=0.8). (**a**)-(**c**) The prevalence of MRSA colonization and infection among patients. (**d**)-(**f**) The prevalence of MRSA infection among patients

To explore how the control factors (*σ* and *κ*) that reflect infection prevention and control strategies towards high-risk patients and patients infected with MRSA influence MRSA prevalence in the ICU, both of the control factors are varied as shown in Fig. 3a-f. It can be clearly seen that the control factor related to targeted control measures towards high-risk patients plays a greater role in reducing MRSA prevalence in patients as compared to the control factor related to targeted control measures towards infected patients. In addition, the trend does not change even if the proportion of high-risk patients at admission increases.

According to Table 2, the most sensitive parameter is the proportion of high-risk patients in the ICU ($\omega _{\theta }^{C_{L}, I_{L}}\approx -2$). Other important parameters include the proportions of colonized patients at admission of the high-risk and low-risk groups, treatment rates, discharge rates, decontamination rate of HCWs, transmission coefficient from colonized patients to uncontaminated HCWs, numbers of patients, and control factor related to targeted measures on high-risk patients. In addition, the sensitivity indices suggest that MRSA prevalence among patients in the high-risk group significantly increases according to the higher proportion of colonized patients of the high-risk group at admission $(\omega _{\lambda _{CH}}^{C_{H}, I_{H}}=1.2227, 1.0608)$. For the higher prevalence of MRSA infection, it may also involve some other factors such as a higher discharge rate, the higher probability of developing an MRSA infection, and a lower treatment rate in high-risk patients $\left (\omega _{\gamma _{H}, p_{H}, \nu _{H}}^{I_{H}}=1.0064, 0.9675, -1.0002\right)$. The sensitivity indices also suggest that the higher proportion of colonized patients at admission, a lower discharge rate, a lower treatment rate, and the higher probability of developing an MRSA infection of low-risk patients may result in higher MRSA prevalence in the low-risk group $(\omega _{\lambda _{CL}}^{C_{L}, I_{L}}=0.9766, 0.7107, \omega _{\gamma _{L}, \nu _{L}}^{C_{L}}=-1.0089, -1.0001, \omega _{\nu _{L}, p_{L}}^{I_{L}}=-1.0001, 0.7601)$. Moreover, there are also some factors that may lead to the higher prevalence of MRSA in the low-risk group: the lower proportion of high-risk patients and the higher discharge rate of high-risk patients $(\omega _{\theta }^{C_{L}, I_{L}}=-2.0023, -2.0010, \omega _{\gamma _{H}}^{C_{L}, I_{L}}=1.0024, 1.0015)$. Finally, the sensitive indices suggest that the higher proportion of colonized patients in the high-risk group at admission and the higher transmission coefficient from colonized patients to uncontaminated HCWs may result in the higher prevalence of HCW contamination $(\omega _{\lambda _{CH},\alpha _{C}}^{H_{C}}=1.0293,0.8590)$. Also, decreasing decontamination rate, control factor related to high-risk patients, and numbers of patients may significantly increase the higher prevalence of HCW contamination $(\omega _{\sigma,\eta,N_{p}}^{H_{C}}=-0.8254, -1.0028, -0.8738)$.

## Discussion

Despite advances in infection prevention and control interventions and successful control strategies, MRSA still continues to be a challenging problem causing morbidity and mortality in the ICU patients. Currently, to reduce the spread of MRSA, many control strategies have been proposed. Some of those are for example screening patients on admission to the ICUs, implementing a contact precaution policy, isolating and cohort nursing MRSA-positive patients, decolonizing MRSA in colonized and infected patients, and limiting use of antibiotics.

In this study, a mathematical model was developed to investigate MRSA transmission among high-risk and low-risk patients via hands of HCWs, study the influences of high-risk patients in an ICU, and explore control strategies. Previous studies have demonstrated that advanced age, MRSA colonization, use of invasive devices, prolonged length of stay, and serious underlying illness are for example possible risk factors for MRSA colonization and infection[15–18]. As active surveillance and stringent control measures for all patients can be financially challenging especially for large health care institutions and can possibly impose a heavy burden on nursing time, some institutions recommend targeted surveillance and control [28, 29, 50]. Based on the model, we explored the effects of targeted control strategies and possible ways to reduce MRSA prevalence.

The basic reproduction number was calculated and it is approximately 0.55 in our baseline result. The result corresponds to one approximated in a preceding study [30] but is less than 1.52 approximated in another study [13]. Although, the basic reproduction number is less than 1, MRSA still persists in the ICU patients due to the presence of MRSA-positive patients at admission. In this present study, MRSA prevalence is approximately 25% which leads to the acquisition rate of 10% in our baseline result. Both approximations are in possible ranges of MRSA prevalence and the acquisition rate [9]. Generally, the basic reproduction number can be used for not only determining whether a pathogen can persist in a population but also investigating the prevalence and severity of outbreaks. Consequently, our results suggest that there are several factors that may influence MRSA prevalence. Those factors are such as a ratio of high-risk to low-risk patients, transmission coefficients, control factors, a ratio of patients to HCWs, and treatment rates.

We further investigated the effects of high-risk patients and other control parameters on MRSA prevalence by numerical simulations. Our results suggest that the higher prevalence of MRSA is linked to the proportion of high-risk patients in an ICU, the presence of colonized and infected patients in the high-risk group, the probability of developing an MRSA infection, and a control factor associated with high-risk patients. These results also correspond to a formula of the basic reproduction number. For example, the control factor related to targeted control measures towards high-risk patients is linked to a squared term in the basic reproduction number’s formula while a control factor associated with infected patients is linked to a simple term. This corresponds to a higher impact of *σ* over *κ* on MRSA prevalence in our numerical results.

In order to explore the influences of parameters in the model, sensitivity analysis was performed. It was found that the proportion of high-risk patients in an ICU, the proportion of colonized patients at admission, treatment rates, discharge rates, decontamination rate of HCWs, transmission coefficients, and the probability of developing an MRSA infection in high-risk patients play an important role in determining MRSA prevalence in the ICU. The increasing or decreasing values of those quantities may result in the higher or lower prevalence of MRSA colonization and infection. For example, our results suggest that the higher proportion of colonized patients in each patient group at admission may lead to higher MRSA prevalence in the patient group. Hence, reducing MRSA colonization and infection on admission may dramatically lower MRSA prevalence. In addition, according to the sensitivity indices, if the probability of developing an MRSA infection in high-risk patients is high, it may result in the higher prevalence of MRSA infection. As transmission coefficients and decontamination rate also significantly affect MRSA prevalence in our sensitivity analysis and they are linked to standard control measures, the results underline how important standard measures can help reduce the prevalence. Although those aforementioned factors may play an important part in determining overall MRSA prevalence, it is unfortunate that some are not manageable and controllable. However, if there are opportunities for control improvement, our results suggest a control effort towards high-risk patients. This results from the sensitivity indices demonstrating that the control factor reflecting targeted control measures towards infected patients (*κ*) shows a smaller impact on reducing the prevalence of MRSA in comparison to the control factor reflecting targeted control measures towards high-risk patients (*σ*). As the latter significantly helps reduce MRSA contamination in HCWs, it may consequently decrease cross-transmission among patients via hands of HCWs. In conclusion, our results from the basic reproduction number, numerical simulations, and sensitivity analysis are in agreement one another. They also correspond to preceding studies that suggest targeted surveillance and decolonization as a possible way to control MRSA with more efficient use of resources [29, 51, 52].

There are several limitations in this study. Firstly, we made an assumption that colonized and infected patients cannot become fully uncolonized in the model which is not in agreement with some preceding reports that suggest a much lesser extent of MRSA in ICU patients [53, 54] and hence it may lead to an overestimate of MRSA prevalence. Secondly, an assumption that the probability of successful colonization and the average contact number between HCWs and patients are not different between high-risk and low-risk patients may also result in an overestimate of MRSA prevalence in low-risk patients. Thirdly, we did not take into account MSSA which may have a potential impact on MRSA prevalence. As hospitals may have different control strategies and some ICUs may have different endemic rates of MRSA and different characteristics, our findings only suggest possible results for some ICU settings. Other scenarios such as a highly endemic setting or a lower endemic setting that affects ranges of parameters may need further investigations.

All in all, we believe that this study will help emphasize the influences of high-risk patients in an ICU, suggest important factors of the MRSA prevalence, and highlight the positive effects of control strategies towards high-risk patients.

## Conclusions

The findings of this study suggest that the proportion of high-risk patients in an ICU and the proportion of colonized and infected patients in the high-risk group at admission may play an important role on MRSA prevalence. In addition, control strategies that significantly help reduce MRSA cross-transmission in high-risk patients may help reduce overall MRSA prevalence.

## Data Availability

All data generated or analysed during this study are included in this published article.

## Abbreviations

HCW: Health care worker
ICU: Intensive care unit
LOS: Length of stay
MRSA: Methicillin-resistant *Staphylococcus aureus*
